# Arsenite-loaded nanoparticles inhibit PARP-1 to overcome multidrug resistance in hepatocellular carcinoma cells

**DOI:** 10.1038/srep31009

**Published:** 2016-08-03

**Authors:** Hanyu Liu, Zongjun Zhang, Xiaoqin Chi, Zhenghuan Zhao, Dengtong Huang, Jianbin Jin, Jinhao Gao

**Affiliations:** 1 State Key Laboratory of Physical Chemistry of Solid Surfaces, The Key Laboratory for Chemical Biology of Fujian Province, and Department of Chemical Biology, College of Chemistry and Chemical Engineering, Xiamen University, Xiamen 361005, China; 2 Fujian Provincial Key Laboratory of Chronic Liver Disease and Hepatocellular Carcinoma, Zhongshan Hospital, Xiamen University, Xiamen 361004, China

## Abstract

Hepatocellular carcinoma (HCC) is one of the highest incidences in cancers; however, traditional chemotherapy often suffers from low efficiency caused by drug resistance. Herein, we report an arsenite-loaded dual-drug (doxorubicin and arsenic trioxide, *i*.*e*., DOX and ATO) nanomedicine system (FeAsO_x_@SiO_2_-DOX, Combo NP) with significant drug synergy and pH-triggered drug release for effective treatment of DOX resistant HCC cells (HuH-7/ADM). This nano-formulation Combo NP exhibits the synergistic effect of DNA damage by DOX along with DNA repair interference by ATO, which results in unprecedented killing efficiency on DOX resistant cancer cells. More importantly, we explored the possible mechanism is that the activity of PARP-1 is inhibited by ATO during the treatment of Combo NP, which finally induces apoptosis of HuH-7/ADM cells by poly (ADP-ribosyl) ation suppression and DNA lesions accumulation. This study provides a smart drug delivery strategy to develop a novel synergistic combination therapy for effectively overcome drug- resistant cancer cells.

Hepatocellular carcinoma (HCC) is one of the most common cancers all over the world, especially in Asian countries^1^. The traditional approach for patients with advanced HCC is chemotherapy. Nevertheless, HCC develops drug resistance very easily to most conventional chemotherapeutic agents. Clinical trials have shown that sorafenib, a conventional chemotherapeutic drug for patients with advanced HCC, could only prolong median survival by nearly 3 months more than those given placebo^2^. Several studies found that the mechanism of HCC drug resistance is complicated, and probably is a combination of two aspects: on one hand, drug delivery to cancer cells is impaired, which generally results from overexpression of ATP-binding cassette (ABC) transporters such as P-glycoprotein (P-gp); on the other hand, drug sensitivity is affected by various changes in drug resistant cancer cells, including increased repair of DNA damage, reduced apoptosis, and altered metabolism of drug^3^,^4^,^5^. In response to these problems, combination therapy have been developed and used widely in many malignant diseases, such as AIDS and cancers^6^. Unfortunately, conventional synergistic therapy is limited by different pharmacokinetics, biodistributions, and membrane transport properties among various drug molecules, which results in difficult optimization for drug ratios^7^. Moreover, conventional synergistic therapy may induce synergistic side-effect, which limits synergistic combinations to narrower biological contexts compared to single drugs^8^. Therefore, it is crucial to develop a new strategy for combination therapy to treat drug resistant HCC.

The rising of nanotechnology has provided a versatile platform for cancer treatment. Because of their large surface-to-volume ratio, high flexibility for surface tailoring, and excellent capacity for multifunction^9^, nanoparticles have recently emerged as a promising carrier for co-delivery of multiple drugs with many advantages including improved drug solubility^10^, controllable release of drugs^11^, precise ratiometric drug loading for synergy^12^, and reduced systemic toxicity^13^, which provides a potential solution for the problems of conventional combination therapy. For example, doxorubicin (DOX), a common chemotherapeutic drug in clinical practice, can adversely stimulate cancer cells to overexpress P-gp, activate DNA repair, increase glutathione transferase activity, and eventually lead to drug resistance^14^. To overcome DOX resistance, several nanoparticle-assisted combination therapies have been developed, such as co-delivery of DOX and other chemotherapeutic drugs^15^,^16^, co-delivery of DOX and P-gp inhibitors^17^,^18^, and co-delivery of DOX and siRNA^19^,^20^. However, most of studies paid more attention to preventing drug efflux in drug resistant cells and enhancing drug accumulation instead of disrupting other means that cells might use to resist drugs, such as increasing repair of DNA damage, reducing apoptosis and altering drug metabolism. As a result, the potential of combination therapy is far from being fully exploited.

Arsenic trioxide (ATO, As_2_O_3_), a drug approved by U.S. Food and Drug Administration (FDA) for clinical treatment of acute promyelocytic leukemia (APL), also has a promising therapeutic effect on solid tumors^21^,^22^. The toxicity of ATO is likely due to the high affinity of trivalent arsenic species with sulfhydryl groups, which can displace Zn^2+^ in zinc fingers of cellular cysteines-containing proteins^23^. Binding of arsenic to these proteins, such as PML-RARα (an oncogenic protein in acute promyelocytic leukemia)^24^ and poly(ADP-ribose) polymerase-1 (PARP-1, a nuclear protein enzymes involved in DNA damage response)^25^,^26^, could alter their conformations and functions as well as their interactions with other functional proteins. Therefore, ATO has been utilized as a PARP-1 inhibitor for combination therapy with DNA-damaging treatment, such as radiation therapy^27^. Unfortunately, poor bioavailability and undesirable side effects, such as skin reactions and liver dysfunction, limits the clinical applications of ATO in treatment of solid tumors^28^. Our previous studies have shown that a suitable carrier could improve the efficacy of ATO to solid tumor treatments and reduce side effects^29^,^30^,^31^. Recently, researchers found that co-delivery of DOX and As^3+^ in a polymer could overcome drug resistant breast cancer, although the mechanism is still unclear^32^. Thus nanoparticles loaded with a DNA damage inducer (*e*.*g*., DOX) and a DNA damage repair inhibitor (*e*.*g*., ATO) is an attractive strategy to kill drug-resistant cancer cells.

Herein, we establish a simple pH-sensitive silica nanoparticles system (Combo NP) with efficient loading of DOX and inorganic nanocomposites [Fe(HAsO_3_)]_n_ (ATO prodrug) with controllable molar ratios based on our previous reports^29^,^30^,^31^,^33^. This smart Combo NP enables increased accumulation and controlled release of drugs (DOX and ATO) in DOX resistant HuH-7/ADM cells, which successfully inhibits the activity of DNA repair protein PARP-1 and induces cell apoptosis. More importantly, promotion of synergistic actions and prevention of drug resistance development in cancer cells by this combination therapeutic nanomedicine indicates that our novel dual-drug system is a promising strategy in cancer treatment.

## Results and Discussion

### Preparation and characterization of Combo NP

**Figure 1:**
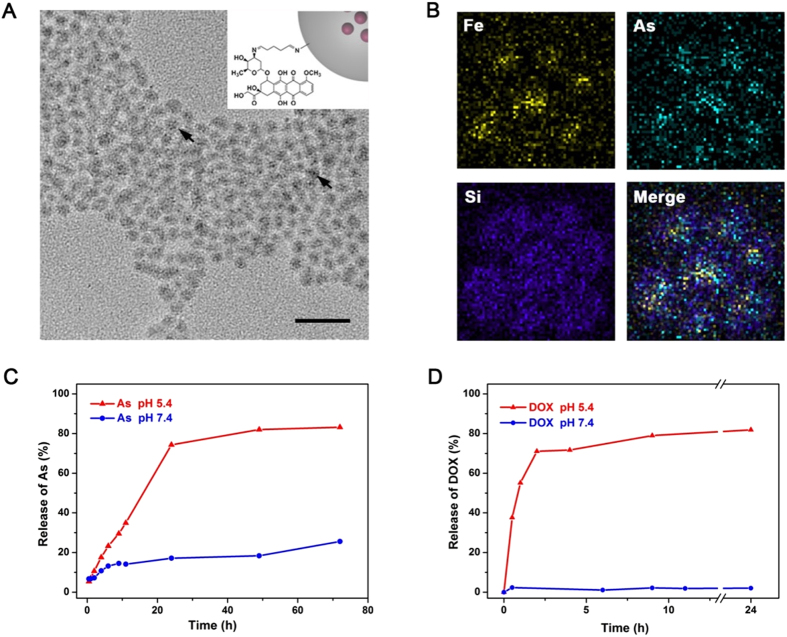
Characterization of Combo NP. (**A**) TEM image of Combo NP. Scale bar, 50 nm. Insert: Schematic illustration of Combo NP structure described in this article. (**B**) EDX mapping images of Combo NP, indicating the co-existence of Fe and As in SiO_2_ shell. Releasing profiles of (**C**) As and (**D**) DOX from Combo NP at different pH values.

The nano-size ATO prodrug was obtained by deposition of arsenite with transitional metal ions (*e*.*g*., Fe^2+^) in reverse micelles^31^, followed by *in-situ* coating with amine-functionalized silica nanoshell (FeAsO_x_@SiO_2_-NH_2_). DOX molecules were anchored on FeAsOx@SiO_2_-NH_2_ nanoparticles by imine bonds, a pH-sensitive cross-linker, to form dual-drug loaded nanomedicine system (FeAsO_x_@SiO_2_-DOX, Combo NP). The different amounts of amine moieties on the surface brought varied DOX/ATO molar ratios (*e*.*g*., 1:1, 1:2 and 1:4), which were determined by fluorometry (emission at 590 nm) and inductively coupled plasma mass spectrometry (ICP-MS). Transmission electron microscopy (TEM) image shows highly uniform morphology of the as-prepared Combo NP (Fig. 1A). Although varied numbers of cores are trapped in silica shells, ATO-based nanocomplexes are clearly observed inside silica (marked by arrows in Fig. 1A). X-ray spectroscopy (EDS) mapping images indicate the presence of iron and arsenic atoms in the SiO_2_ shell, which further confirms the successful fabrication of core-shell nanostructure (Fig. 1B). Dynamic light scattering (DLS) analysis shows that the average size of Combo NP is 12.0 ± 1.3 nm with a narrow distribution (Supplementary Fig. S1). The Combo NP exhibits a positive zeta potential at about +43.2 mV (Supplementary Fig. S1) due to the anchored DOX molecules and a few remained amine moieties on the silica surface. Since pH variations have been exploited to control drug delivery in mildly acidic microenvironment of tumors as well as intracellular compartments (such as endosomes or lysosomes)^34^,^35^,^36^, Combo NP is also expected to have a capability for pH-triggered release of two drugs. The releasing processes of arsenic and DOX in different pH environments (5.4 and 7.4) were monitored by ICP-MS and fluorescence spectrophotometry, respectively. As showed in Fig. 1C, 74.4% and 17.1% of loaded arsenic were released within 24 h incubation at pH 5.4 and 7.4, respectively. By comparison, DOX showed a fast release in the acidic condition (pH 5.4), and the release rate could be up to 79.0% within 9 h and then reach to 81.9% after 24 h (Fig. 1D). However, DOX was found to be slightly released (up to 2.05%) in the neutral environment (pH 7.4, similar to plasma) after 24 h incubation, indicating the good stability of Combo NP in blood circulation and healthy tissues. These results suggest that the Combo NP is stable under neutral conditions while sensitive to acidic environment, which is highly desirable for tumor therapy.

### Cytotoxicity and synergism of Combo NP

**Figure 2:**
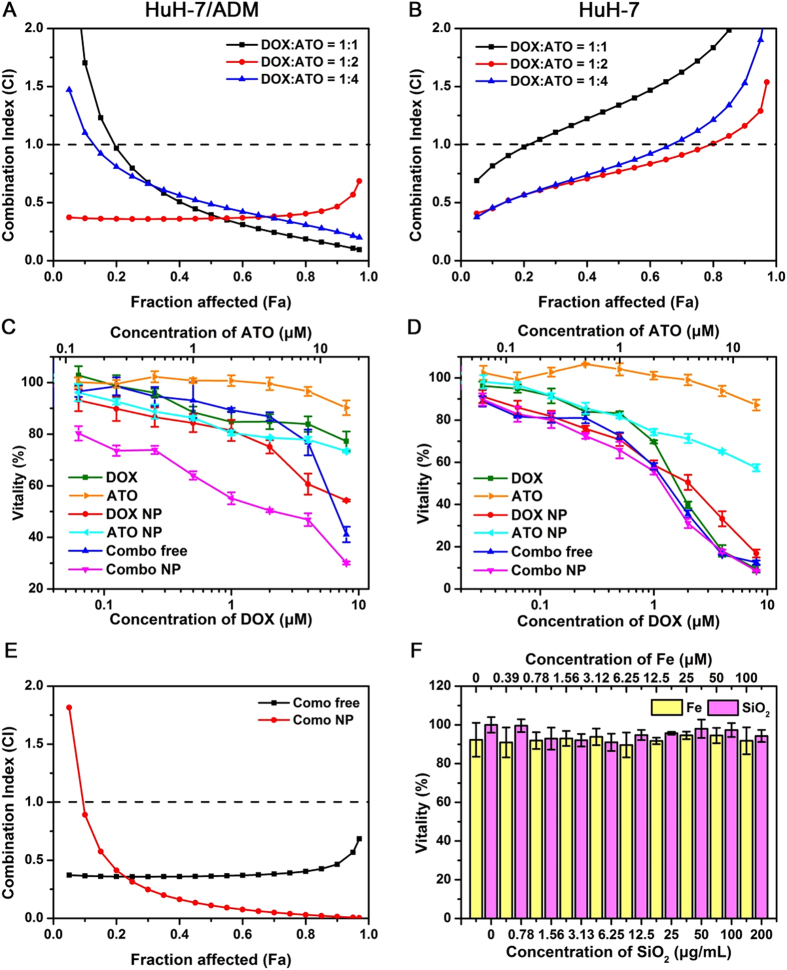
Cytotoxicity and synergy. Trends of Combination Index (CI) with different ratiometric mixtures of DOX and ATO for (**A**) HuH-7/ADM cells and (**B**) HuH-7 cells. Vitality of (**C**) HuH-7/ADM and (**D**) HuH-7 after treated with different drug formulations at various concentrations for 24 h. (**E**) Comparison of CI values after treatments of Combo free and Combo NP in HuH-7/ADM cells. The molar ratio of DOX and ATO was fixed at 1:2. (**F**) Vitality of HuH-7/ADM treated with Fe ions or SiO_2_ for 24 h. All data are represented as average ± standard deviation (*n* = 5).

To demonstrate the synergistic effect of DOX and ATO *in vitro*, we firstly tested the cytotoxicity of free DOX and ATO in wild-type HuH-7 and DOX-resistant HuH-7/ADM cells for 24 h (Supplementary Fig. S2 and Table 1). We found that HuH-7/ADM cells were highly resistant to DOX. The IC_50_ value of DOX in HuH-7/ADM cells (172.90 ± 12.06 μM) was over 100-fold higher than that in parent HuH-7 cells (1.70 ± 0.10 μM). Several studies have reported that ATO with low concentration can enhance the cytotoxicity of other drugs (*e*.*g*., cisplatin^37^ and L-buthionine-sulfoximine^38^,^39^) or UV radiation^40^. We further investigated the synergistic effect of combined free DOX with ATO according to the isobologram equation of Chou-Talalay, which could provide the combination index (CI) to quantitatively evaluate the interactions of drugs: synergism (CI < 1), additive effect (CI = 1), and antagonism (CI > 1)^41^. To keep the dosing of ATO in an extremely low range, we compared the cytotoxicity of DOX/ATO mixture in varied proportions, including molar ratio on 1:1, 1:2, and 1:4. Among these variations, minimal CI value was obtained when the molar ratio of DOX/ATO was 1:2 for both HuH-7 and HuH-7/ADM cells (Fig. 2A,B). Particularly, there was a strong synergistic effect (CI value < 0.5) in HuH-7/ADM cells at 1:2 ratio of DOX/ATO when fraction affected (Fa) value was in the validated range of 0 to 0.9 (Fig. 2A). Therefore we fixed the drug molar ratio of DOX/ATO as 1:2 for further experiments.

We then evaluated cytotoxicity of various drug formulations with the same doses in HuH-7 and HuH-7/ADM cells for 24 h, including free DOX, free ATO, DOX-loaded SiO_2_ nanoparticles (DOX NP), ATO-loaded SiO_2_ nanoparticles (ATO NP), combination of free DOX and ATO (Combo free) and dual-drug loaded SiO_2_ nanoparticles (Combo NP). The IC_50_ values were listed in Table 1. Among all treatments, Combo NP was the best at killing DOX-resistant HuH-7/ADM cells with the IC_50_ value of 2.2 ± 0.05 μM, indicating that the drug activity of Combo NP was about 80 times higher than that of free DOX (Fig. 2C and Table 1). Specifically, HuH-7/ADM cells were highly resistant to free DOX even with a dose up to 8 μM. The cytotoxicity of DOX NP was raised by 14.46-fold (IC_50_ = 11.96 ± 1.55 μM), which might be owing to high cellular uptake of DOX NP. It is interesting that although the activity of Combo free was similar to free DOX at low concentrations (~4 μM), the cytotoxicity was strengthened significantly when the concentrations of DOX and ATO go up. Indeed, ATO with the concentrations used for combination therapy (0.06 to 16 μM) was nontoxic, suggesting that ATO might enhance the therapeutic effect of DOX in HuH-7/ADM cells via a synergistic manner. Compared with Combo free, Combo NP exhibits better synergistic effect when Fa value is from 0.2 to 1.0 (Fig. 2E) in HuH-7/ADM. For HuH-7 cells, the cytotoxicity of various drug formulations is highly comparable (Fig. 2D and Table 1). We found that the IC_50_ values of Combo NP to HuH-7/ADM (2.20 ± 0.05 μM) and HuH-7 cells (1.24 ± 0.02 μM) are close. We also tested the cytotoxicity of different drug formulations in HuH-7 and HuH-7/ADM cells for 48 h (Supplementary Fig. S2), and the IC_50_ of Combo NP had further decreased to 0.53 μM. The similarity of trends suggests that Combo NP can overcome drug resistance of tumor cells effectively. It should be noted that SiO_2_ nanocarriers or Fe ions did not have any significant cytotoxicity even at the concentration of up to 200 μg/mL or 100 μM, respectively (Fig. 2F).

### Drugs accumulation and subcellular localization

**Figure 3:**
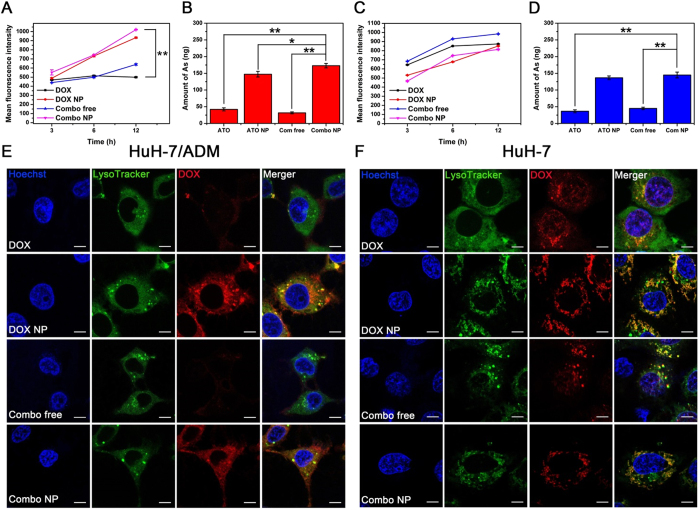
Cellular drug accumulation and localization. Flow cytometry analysis of DOX fluorescence in (**A**) HuH-7/ADM and (**C**) HuH-7 cells after incubation with DOX, DOX NP, Combo free and Combo NP (the concentrations of DOX were all 2 μM). ICP-MS analysis of As in (**B**) HuH-7/ADM cells and (**D**) HuH-7 cells after incubation with ATO, ATO NP, Combo free and Combo NP (the concentrations of ATO were all 4 μM) for 12 h. All data are represented as average ± standard deviation (*n* = 3), *p < 0.05; **p < 0.01. Confocal fluorescence imaging of (**E**) HuH-7/ADM cells and (**F**) HuH-7 cells treated with DOX, DOX NP, Combo free, and Combo NP (the concentrations of DOX were all 4 μM) for 6 h, scale bars: 7.5 μm. Hoechst 33342 and LysoTracker green were used to stain cell nuclei and lysosome, respectively.

Cancer cells can acquire drug resistance by overexpression of efflux proteins (*e*.*g*., P-gp), which can significantly reduce effective concentrations of drug in cells^3^,^4^,^5^. We confirmed that DOX-resistant HuH-7/ADM cells overexpressed P-gp by Western blotting and immunofluorescence (Supplementary Fig. S3). To understand the mechanisms underlying of enhanced therapeutic effect in Combo NP, we set out to study intercellular drug accumulation. After incubation with different drug formulations, cellular fluorescence intensities of DOX were measured by flow cytometry (Fig. 3A,C). For HuH-7/ADM cells, intracellular accumulation of free DOX was low, as expected, and the DOX accumulation slightly decreased after incubation for 12 h (Fig. 3A), probably due to the efflux effect of P-gp. However, much higher accumulation of DOX was observed after incubation with DOX NP or Combo NP, and fluorescence intensities were nearly 2 times higher than that under free DOX treatment. On the contrary, for HuH-7 cells, free DOX could enter cells rapidly and result in substantial intracellular drug accumulation (Fig. 3C). Although nanocarriers accumulated in HuH-7 cells more slowly than free drugs, the DOX fluorescence intensities of all groups were comparable after incubated with drugs for 12 h. Remarkably, the fluorescence intensity of DOX somewhat increased after Combo free treatment in both HuH-7 and HuH-7/ADM cells, which might be a result of increased permeability of cell membrane caused by apoptotic promotion of ATO, so that a large number of free DOX could be internalized into cells. This cellular uptake of DOX was further confirmed by confocal laser scanning microscopy (Fig. 3E).

We also quantitatively determined the cellular amount of As by ICP-MS in both HuH-7/AMD and HuH-7 cells (Fig. 3B,D). It appeared that free ATO-treated cells absorbed a tiny bit of As because the uptake of free ATO probably needs the assistance of transport proteins^42^, which might protect cells from arsenic poisoning. However, remarkable accumulations of As were detected in both HuH-7 and HuH-7/ADM cells when treated with ATO NP and Combo NP. The amounts of As in ATO NP- or Combo NP-treated groups were nearly 3-fold higher than those incubated with free ATO and Combo free. This phenomenon might be attributed to the positive zeta potential of nanoparticles that is of benefit to the cellular internalization. These observations suggest that DOX NP, ATO NP and Combo NP can increase drug accumulations in drug resistant cells.

When nanoparticles encounter cells, they will be internalized into the cells through several endocytosis pathways^43^, and then transferred to various organelles, such as lysosomes, Golgi apparatus, mitochondria, and nucleus. During these processes, it would be better if nanoparticles could be degraded or disassembled to quickly release their payload^44^. To further verify our assumption that drug-loaded Combo NP could be efficiently internalized into drug resistant cells and localized in the acidic organelles to release drugs, we compared the cellular uptake behaviors of free DOX, Combo free, DOX NP, and Combo NP in HuH-7/ADM (Fig. 3E) and HuH-7 cells (Fig. 3F). HuH-7/ADM cells treated with free DOX or Combo free showed low signals after incubated for 6 h. However, after incubated for 12 h, Combo free could dramatically improve the uptake of DOX due to the addition of ATO (Supplementary Fig. S4). This phenomenon further confirmed that ATO could enhance the uptake of DOX in HuH-7/ADM. On the contrary, the internalization efficiencies for DOX NP and Combo NP were much higher. The DOX fluorescence was mainly localize in lysosomes and was also observed in the cytoplasm after incubated for 6 h (Fig. 3E), suggesting that nanocarrier were internalized in lysosomes and then released their cargos in cytoplasm. After incubated for 12 h, we could observe DOX fluorescence in the nucleus (Supplementary Fig. S4), indicating that DOX released from SiO_2_ and exerted anticancer effect. For HuH-7 cells, DOX fluorescence intensities were comparable when treated with different drug formulations, which further confirmed that loading drugs in nanoparticles does not affect the uptake of DOX. Interestingly, free DOX and Combo free could rapidly enter into nucleus in HuH-7 cells (Fig. 3F), while DOX NP and Combo NP could gradual accumulate in the nucleus after 12 h incubation (Supplementary Fig. S4). Based on these findings, it can be concluded that drug-loaded nanoparticles (DOX NP and Combo NP) can significantly enhance the uptake of drugs in HuH-7/ADM and control drugs releasing in acidic organelles.

### DNA damage

**Figure 4:**
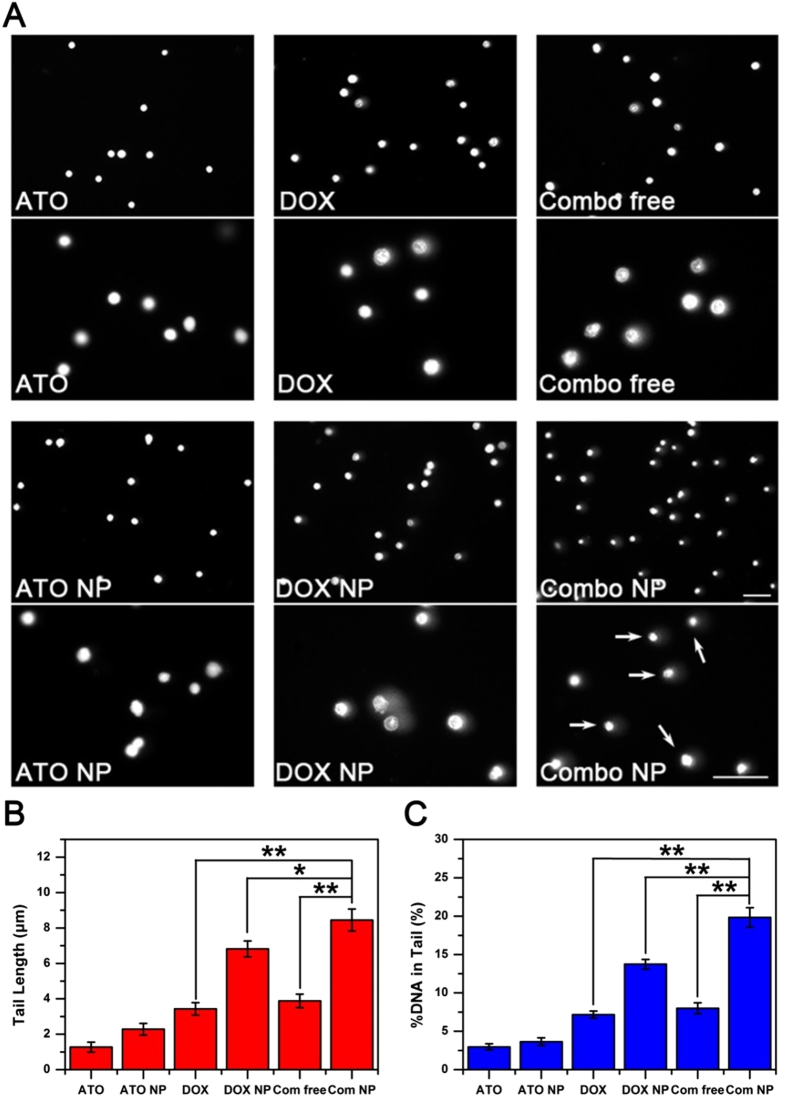
Comet assay for DNA damage. (**A**) Fluorescence microscope imaging of HuH-7/ADM cells treated with different drug formulations (2 μM DOX and 4 μM ATO) for 12 h. The two pictures (upper and lower) were taken in the same experiment with different magnifications. DNA was stained by PI, scale bars: 50 μm. (**B**) Tail length and (**C**) percentage of DNA in tail were analyzed for 200 cells at random by CometScore software. All data are represented as average ± standard deviation (*n* = 3), *p < 0.05; **p < 0.01.

Chemotherapeutic drugs induce DNA damage either directly (*e*.*g*., platinum-based drugs) or indirectly (topoisomerase inhibitors, such as DOX). One of the mechanisms in cellular drug resistance is the improved ability of DNA damage repair. To this end, we comprehensively investigated DNA damage in HuH-7 and HuH-7/ADM cells after different treatments by comet assay (Fig. 4, Supplementary Fig. S5 and S6). DNA damage was evaluated by tail length and percentage of DNA fragments in comet tail. DOX (2 μM) caused DNA damage in a time dependent manner (Supplementary Fig. S5), while DNA damage caused by ATO alone (4 μM) was negligible (Supplementary Fig. S6). Combo free can significantly enhance the DNA damage in HuH-7 cells. The tail length and the percentage of DNA in the comet tail increase by 1.97- and 2.29-fold compared to DOX-treated groups, respectively (Supplementary Fig. S6). Furthermore, we verified the synergistic effect of Combo free and Combo NP in HuH-7/ADM cells. As shown in Fig. 4, Combo NP exhibited a robust potentiation of DOX-induced DNA damage, which results in almost all cells had comet tails (indicated by arrows in Fig. 4A). The tail length and tail DNA content for Combo NP- treated cells were 2.18- and 1.44-fold more than that of cells treated with Combo free, respectively (Fig. 4B,C). In contrast, neither DOX nor combo free displayed any notable aggravation in DNA damage. DOX NP slightly increased the degree of DNA damage, which might be due to the enhancement of drug accumulation in HuH-7/ADM. These observations suggest that the nanocarriers could enable synergy of DOX and ATO to overcome drug resistance.

**Figure 5:**
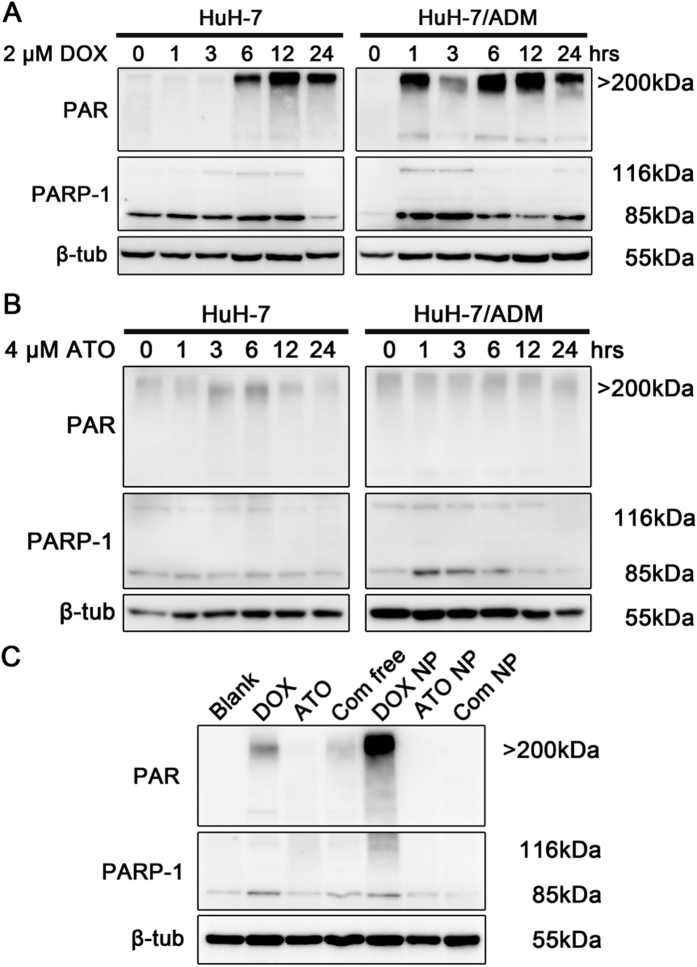
PARP-1 activation after DOX treatment and inactivation after ATO treatment. The expression levels of PAR, PARP-1 and β-tubulin were detected by western blot. All samples were processed under the same experimental conditions. Cropped gels were used to improve the clarity. (**A**) HuH-7 and HuH-7/ADM cells were treated with 2 μM DOX for different incubation times. In response to DNA damage caused by DOX, massive PAR was rapidly generated by PARP-1 in HuH-7/ADM cells. (**B**) HuH-7 and HuH-7/ADM cells treated with 4 μM ATO for different incubation times. HuH-7/ADM cells treated with ATO could not induce the synthesis of PAR. (**C**) HuH-7/ADM cells were treated with different drug formulations (2 μM DOX and 4 μM ATO) for 12 h. DOX and DOX NP induced activation of PARP-1 and catalyzed PAR polymer production. Nevertheless, expression of PAR was dramatically inhibited in the presence of ATO after combinational treatments, especially Combo NP. Full-length blots with multiple exposures are presented in Supplementary Fig. S7.

In response to DNA damage, PARP-1 can be activated immediately, which catalyzes poly(ADP-ribosyl)ation in DNA lesions and induces massive synthesis of ploy(ADP-ribose) (PAR) to facilitate DNA damage repair^45^,^46^; and the signal intensity of PAR indicates activity of PARP-1. Recently, researchers found that PARP-1 enhanced lung adenocanrcinoma metastasis, suggesting lung adenocarcinoma patients might benefit from treatment with PARP-1 inhibitors^47^. Moreover, several drug combinations containing DNA damage drugs and PARP-1 inhibitors are currently being tested in clinical trials^48^, indicating that this is a great strategy to enhance the therapeutic effect. As (III) has been reported to disturb DNA damage repair by inhibiting the activities of PARP-1^49^,^50^. Further western blotting analysis revealed a significantly increased PAR expression in HuH-7/ADM cells within one hour after treatment with DOX (Fig. 5A). Nevertheless, after incubation for 6 hours, PAR was synthesized slowly in HuH-7 cells. These results highlighted that drug-resistant cells can detect DNA damage rapidly and activate the DNA damage repair system simultaneously. For comparison, 4 μM ATO-treated HuH-7 and HuH-7/ADM cells showed negligible PAR synthesis, suggesting that ATO treatment does not activate PARP-1 (Fig. 5B and Supplementary Fig S7). We subsequently investigated the activity of PARP-1 in HuH-7/ADM cells after treated with different drug formulations for 12 h (Fig. 5C and Supplementary Fig S7). The results showed that the expression of PAR was evidently attenuated by the treatment of Combo free or Combo NP, especially Combo NP, even though DOX and DOX NP-treated significantly enhanced PAR polymer production. The PAR expression of Combo NP-treated group was similar to that of control group. It is possible that suppression of PAR synthesis by ATO, a PARP-1 inhibitor, abolishes the recruitment of DNA repair proteins, and synergistically aggravates DNA lesions.

### Apoptosis of drug-resistance cancer cells

**Figure 6:**
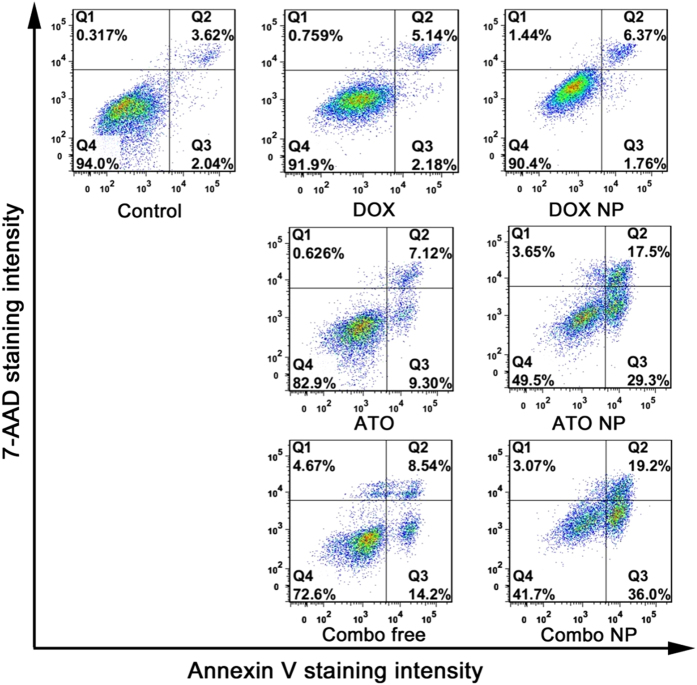
Analysis of cell apoptosis by flow cytometry. HuH-7/ADM cells were treated with different drug formulations (2 μM DOX and 4 μM ATO) for 24 h and stained with 7-aminoactinomycin D (7-AAD, Invitrogen) and Annexin-V (Roche). Combo NP significantly increased the apoptosis of drug resistant cancer cells.

Apoptosis, a gene-directed program, is a mechanism to induce cell death if damaged DNA cannot be repaired^51^,^52^. We next investigated the apoptosis induced by various drug formulations. The traditional method to detect apoptosis is using FITC-Annexin V/propidium iodide (PI) to stain apoptotic cells/necrotic cells, however, the fluorescence spectra of DOX and PI are similar and overlapped in a wide wavelength range. 7-aminoactinomycin D (7-AAD) has similar properties to PI but narrower emission wavelength width and less overlapping with DOX (Supplementary Fig. S8). We therefore chose another dye 7-AAD for staining necrotic cells. In comparison with all drug formulations, cells treated with Combo NP displayed the highest apoptotic rate with up to 55.2% (Fig. 6). In contrast, cells incubated with DOX and DOX NP showed unnoticeable apoptosis, and the ratios of apoptosis cells were only 7.32% and 8.13%, respectively. These results are inconsistent with the observations of Comet assay, which showed that DOX NP could enhance the production of DNA fragments at 12 h in HuH-7/ADM cells. There seemingly contradictory results might be attributed to the fact that drug-resistant cells active PARP-1 to recruit damage-repair proteins for DNA repairing, which finally recover themselves. Slight apoptosis could be observed when cells were exposed to ATO or Combo free with low concentration of ATO (4 μM), which was mostly in agreement with previous reports^53^,^54^. ATO NP could further increase the ratio of apoptosis by enhancing cellular uptake of ATO. In a word, these results indicating that Combo NP can induce apoptosis effectively and overcome drug resistance in HuH-7/ADM cells.

### Possible acting mechanism of dual-drug loaded Combo NP

**Figure 7:**
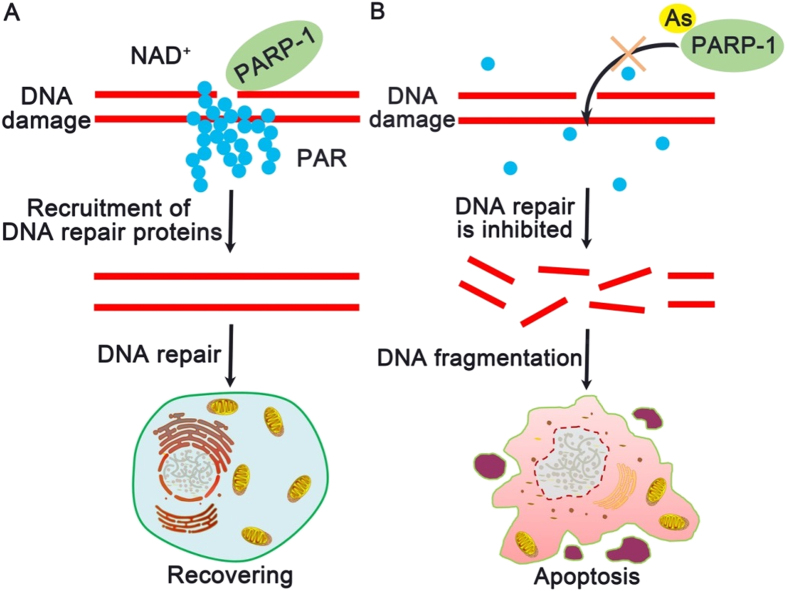
Proposed mechanism of Combo NP mediated alteration to overcome drug resistant in cancer cells. (**A**) In drug resistant cancer cells, PARP-1 is activated rapidly and catalyzes the synthesis of PAR surrounding DNA lesions once DNA damage occurs. DNA repair proteins are recruited by PAR for subsequent repairing, which results in cell recovering. This process causes the failure of traditional chemotherapeutic agents (such as DOX) in cancer treatment. (**B**) As (III), as a PARP-1 inhibitor, displaces Zn^2+^ in the zinc fingers of PARP-1, and disturbs the synthesis of PAR to interfere DNA damage repairing and accumulate DNA fragmentation in cells, which eventually leads to apoptosis.

Based on the above findings, we propose a mechanism of the dual-drug loaded Combo NP for treatment of drug-resistance cancer cells (Fig. 7). Nanocarriers increase intracellular accumulation of cargos (*e*.*g*., DOX and ATO), which causes DNA damage. In the case of administering DOX alone, drug-resistance cancer cells activate PARP-1 to synthesize massive PAR at DNA lesions, facilitate DNA damage repair, and finally recover themselves (Fig. 7A). However, in the case of combinational drugs of DOX and ATO, the binding of ATO to PARP-1 significantly inhibits its activity, which blocks DNA damage repair, increases accumulation of DNA fragments, and finally results in apoptosis of drug-resistant cancer cells (Fig. 7B).

## Conclusions

In summary, we reported a simple method to synthesize a novel dual-drug loaded nanomedicine system for successfully overcoming drug resistance in hepatocellular carcinoma cells with low concentrations of drugs. This smart pH-triggered Combo NP with precisely ratiometric cargos loading could significantly enhance drug accumulation and cytotoxicity of DOX (78.59-fold) in DOX-resistant HuH-7/ADM cells. Furthermore, Combo NP could considerably increase DNA damage caused by DOX through inhibiting the activity of PARP-1, which effectively induced apoptosis. Our results support the notion that dual-drug loaded nanomedicine systems combining DNA-damaging with repair-blocking agents can thoroughly overcome drug resistance in cancer cells. The nanocarriers with small size (~12 nm) and pH-sensitive property have a great potential for tumor therapy *in vivo* due to the enhanced permeability and retention (EPR) effect and tumor acidic microenvironment^44^,^55^. Considering the complexity of physiological environment, our dual-drug loaded nanomedicine systems may need further surface modification and functionalization to achieve a desired *in vivo* biodistribution, which is ongoing. Overall, this study and future work with mouse models should help shed more light on the real potential of new combinational therapy in nanomedicine using ATO as a PARP-1 inhibitor with DNA-damaging drugs for treatment of multiple drug resistant cancers.

## Methods

### Materials

As_2_O_3_ (90%), (3-aminopropyl)triethoxysilane (APTES, 97%), tetraethylorthosilicate (TEOS 99.9%), Iron (II) acetate (tech 97%), Polyoxyethylene(5)nonylphenyl ether (Co-520) were purchased from Alfa Aesar. Sodium metasilicate nonahydrate, ammonium hydroxide, cyclohexane and ethanol were purchased from Sinopharm Chemical Reagent Co. Ltd. (Shanghai, China). Doxorubicin (DOX) was purchased from HuangFeng United Technology Co. Ltd. (Beijing, China). All chemicals were used as received without further purification.

### Synthesis of Combo NP

Following the microemulsion method^56^, FeAsOx nanoparticles were synthesized by precipitation of iron acetate with aqueous ATO (1:1) in cyclohexane (including 29 vol % Igepal Co-520) for 6 h, followed by reaction with TEOS (600 μL, direct addition) overnight. Different amounts of APTES (25, 50, 75 μL) were added to obtain FeAsO_x_@SiO_2_ nanocomposites with different degrees of amine-functionalization (FeAsO_x_@SiO_2_-NH_2_, ATO NP).

Glutaraldehyde was used to transform the terminal groups of nanocomposites from amine to aldehyde. In detail, FeAsO_x_@SiO_2_-NH_2_ nanocomposites with different amount of amine groups on the surfaces were mixed with glutaraldehyde (5.2%, w/v) in PBS and the mixture was stirred for 2 h. After purification, DOX (1 mg, 1.7 mmol) in PBS was added into the mixture and the resulting mixture was stirred for 2 h to form FeAsO_x_@SiO_2_-DOX (Combo NP) with varied molar ratios of DOX/ATO.

Size distribution and zeta potential of Combo NP were measured by Dynamic light scattering (DLS) using a Malvern Zetasizer nano ZS instrument. The morphologic examination of nanocomposites was performed on a JEM-2100 microscope at an accelerating voltage of 200 kV. The element mapping analysis was performed on a Tecnai F30 microscope at an accelerating voltage of 300 kV. The release profile of DOX and As from SiO_2_ nanocarriers was measured in 0.1 M PBS buffer (pH = 7.4) and 0.1 M citric acid buffer solution (pH = 5.4) at 37 °C. At predetermined time points, a certain volume of solution was centrifuged (14000 rpm, 20 min) to collect the supernatant and analyze the releasing profiles (As, DOX). The amount of loaded and released DOX was measured using fluorescence spectrophotometry (HORIBA FL-3000/FM4-3000), and As was detected by ICP-MS.

### Cell culture

Cells were cultured in Dulbecco’s Modified Eagle’s Medium (DMEM medium), containing 10% fetal bovine serum (FBS, Hyclone) and antibiotics (100 mg/mL streptomycin and 100 U/mL penicillin) at 37 °C using a humidified 5% CO_2_ incubator.

### Cytotoxicity assay

HuH-7 and HuH-7/ADM cells were seeded in 96-well plate with the concentration of 5 × 10^4^ cells per well overnight, and then treated with fresh DMEM medium (supplemented 10% fetal bovine serum) containing various concentrations of drugs with different formulations for 24 h. Then culture mediums were replaced by fresh DMEM medium containing 0.5 mg/mL of MTT and the cells were further incubated for 4 h. The mediums were removed, and DMSO was added to dissolve the formazan produced by living cells. Absorbance at 492 nm of each well was measured by MultiSkan FC microplate reader (Thermo scientific). The experiment was performed in triplicate.

### Synergism evaluation

The combination index (CI) and fraction affect (Fa) were put forward to evaluate the synergism. The CI equation is based on the multiple drug-effect equation of Chou-Talalay method^6^,^41^. For each level of Fa, CI values were calculated by CompuSyn software according to the following equation:
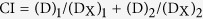


In this equation, D_1_ and D_2_ indicate the doses of drug 1 (DOX) and drug 2 (ATO) in combination that leads to Fa × 100% growth inhibition in the actual experiment, while (D_X_)_1_ and (D_X_)_2_ are the doses of drug 1 and drug 2 alone that results in Fa × 100% growth inhibition.

### Drug accumulation

After treated with different drug formulations (DOX 2 μM and ATO 4 μM) for 3, 6 and 12 h, cells were collected and washed three times to remove unabsorbed drugs. Cellular fluorescence intensities of DOX were measured by flow cytometry. To measure cellular amount of As ions, cells were collected and lysed completely in 500 μL nitric acid (65–68%), and finally fixed volume to 10 mL with DI water. The amount of As ions were detected by ICP-MS.

### Subcellular localization

HuH-7 and HuH-7/ADM cells were seeded in 35 mm confocal plates in DMEM medium supplemented with 10% FBS, and incubated overnight. Then cells treated with fresh medium containing different drug formulations (DOX 4 μM and ATO 8 μM) for 6 h. After washing three times, cells were stained with LysoTracker green (Invitrogen) and Hoechst 33342 (Cell Signaling Technology) for lysosomes and nuclei, respectively. Fluorescent images were taken on a laser scanning confocal microscope (Leica TCS SP5).

### Comet assay

The comet assay was performed using the traditional method^57^,^58^ with appropriate modifications. After treated with different drug formulations (DOX 2 μM and ATO 4 μM), cells were collected and suspended with PBS at the density of 1 × 10^6 ^cells/mL. Then cells were mixed with 1% solution of low melting point agarose at 37 °C, and the mixture was applied to a lid of 96-well plate to form a thin layer (4 °C for 30 min). Cold lysis buffer was added to the lid to lyse cells at 4 °C. After 2 h, the lid was moved into alkaline electrophoresis buffer for 30 min to unwind DNA. Then electrophoresis was carried out at 15 V and 300 mA for 20 min. The lid was washed with neutralizing buffer three times and stained by Propidium Iodide (PI) for 20 min in dark. Comets were recorded by inverted fluorescence microscope (Zeizz, Axio Obserber A1). Tail length and percentage of DNA in tail were analyzed for 200 cells at random by CometScore software.

### Western blotting analysis

After treatment, cells were collected and resuspended in PBS, and mixed with isometric RIPA buffer containing 1% protease inhibitor at 4 °C for 30 min. After centrifuge at 10000 g for 5 min, the supernatant was mixed with equal volume of 2 × loading buffer and heated to 95 °C for 5 min. Proteins were loaded on polyacrylamide gel and run at stock gel (5%) for 15 min (90 V) and at separated gel (8%) for 70 min (130 V). Proteins were resolved on an 8% SDS/PAGE gel and subsequently transferred to a PVDF membrane (Millipore). After blocked with TBS buffer containing 5% skim milk powder (Oxoid) for 30 min, the membrane was incubated in TBS buffer containing 5% milk and primary antibodies against P-gp (1:2500, abcam), PARP-1 (1:1000, abcam), PAR (1:2000, abcam), β-tubulin (1:5000, abcam) for 2 h, respectively, and washed with TBS buffer (5 min × 5). The membrane was further incubated in TBS buffer containing 5% milk and secondary antibodies (1:5000, goat anti-rabbit, Pierce) for 1 h, and washed with TBS buffer (5 min × 5). Bands were visualized by WesternBright ECL (Advansta) and pictures were taken by an imaging system (GE Healthcare Bio-sciences AB). Multi-exposure images were obtained. The expression level of β-tubulin was used as a standard to normalize the expression of other proteins.

### Apoptosis assay

After treated with different drug formulation (DOX 2 μM and ATO 4 μM) for 24 h, cells were collected and washed twice with PBS. Then cells were stained with 7-aminoactinomycin D (7-AAD, Invitrogen) and Annexin-V (Roche) at room temperature for 20 min in dark. The fluorescence was detected by flow cytometry, and the data was analyzed by Flowjo software.

### Statistical analysis

Statistical analysis was performed using the Student’s t-test for unpaired data, p value of less than 0.05 was accepted as a statistically significant difference. Data plotted with error bars are expressed as means with standard deviation (SD), unless otherwise indicated.

## Additional Information

**How to cite this article**: Liu, H. *et al.* Arsenite-loaded nanoparticles inhibit PARP-1 to overcome multidrug resistance in hepatocellular carcinoma cells. *Sci. Rep.* **6**, 31009; doi: 10.1038/srep31009 (2016).

## Supplementary Material

Supplementary Information

## Figures and Tables

**Table 1:** IC_50_ of DOX (24 h) treated with different drug formulations in HuH-7/ADM and HuH-7 cells.

Drug formulation	HuH-7/ADM		HuH-7	
	IC_50_ (μM)	Decreased fold^a^	IC_50_ (μM)	Decreased fold^b^
DOX	172.90 ± 12.06	1	1.70 ± 0.10	1
DOX NP	11.96 ± 1.55	14.46	2.06 ± 0.22	0.82
Combo free	7.00 ± 0.45	24.70	1.36 ± 0.05	1.25
Combo NP	2.20 ± 0.05	78.59	1.24 ± 0.02	1.37
ATO	28.14 ± 0.48		31.24 ± 0.36	
ATO NP	31.59 ± 0.24		25.55 ± 0.46	

^a^Indicates the decreased fold of IC_50_ value compared with free DOX in HuH-7/ADM cells.

^b^Indicates the decreased fold of IC_50_ value compare with free DOX in HuH-7 cells.
