# Supplementary material for: Arsenite-loaded nanoparticles inhibit PARP-1 to overcome multidrug resistance in hepatocellular carcinoma cells
Source: Sci Rep. 2016 Aug 3;6:31009. doi: 10.1038/srep31009 (PMC4971527; doi:10.1038/srep31009)
Supplement: Supplementary Information [file srep31009-s1.pdf]

Supplementary Information

Arsenite-loaded nanoparticles inhibit PARP-1 to overcome multidrug resistance in hepatocellular carcinoma cells

Hanyu Liu,[1,†] Zongjun Zhang,[1,†] Xiaoqin Chi,[2,†] Zhenghuan Zhao,[1] Dengtong Huang,[1] Jianbin Jin,[2] and Jinhao Gao[1]*

[1] State Key Laboratory of Physical Chemistry of Solid Surfaces, The Key Laboratory for Chemical Biology of Fujian Province, and Department of Chemical Biology, College of Chemistry and Chemical Engineering, Xiamen University, Xiamen 361005, China.

[2] Fujian Provincial Key Laboratory of Chronic Liver Disease and Hepatocellular Carcinoma, Zhongshan Hospital, Xiamen University, Xiamen 361004, China.

[†]These authors contributed equally to this work.

[*]Email: jhgao@xmu.edu.cn

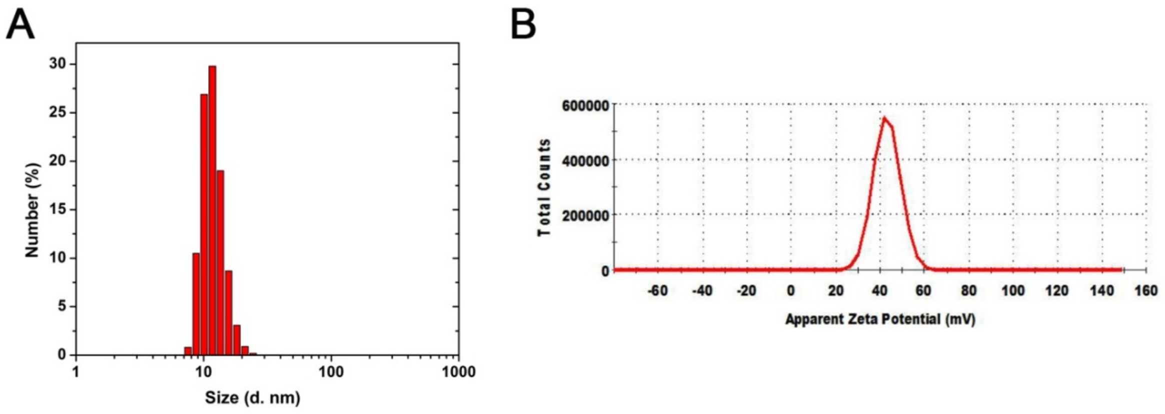

Supplementary Figure 1. (A) Size distribution and (B) Zeta potential analysis of Combo NP determined by DLS. These results show that the average size of Combo NP is 12.0 ± 1.3 nm with a narrow distribution, and zeta potential of Combo NP is about + 43.2 mV.

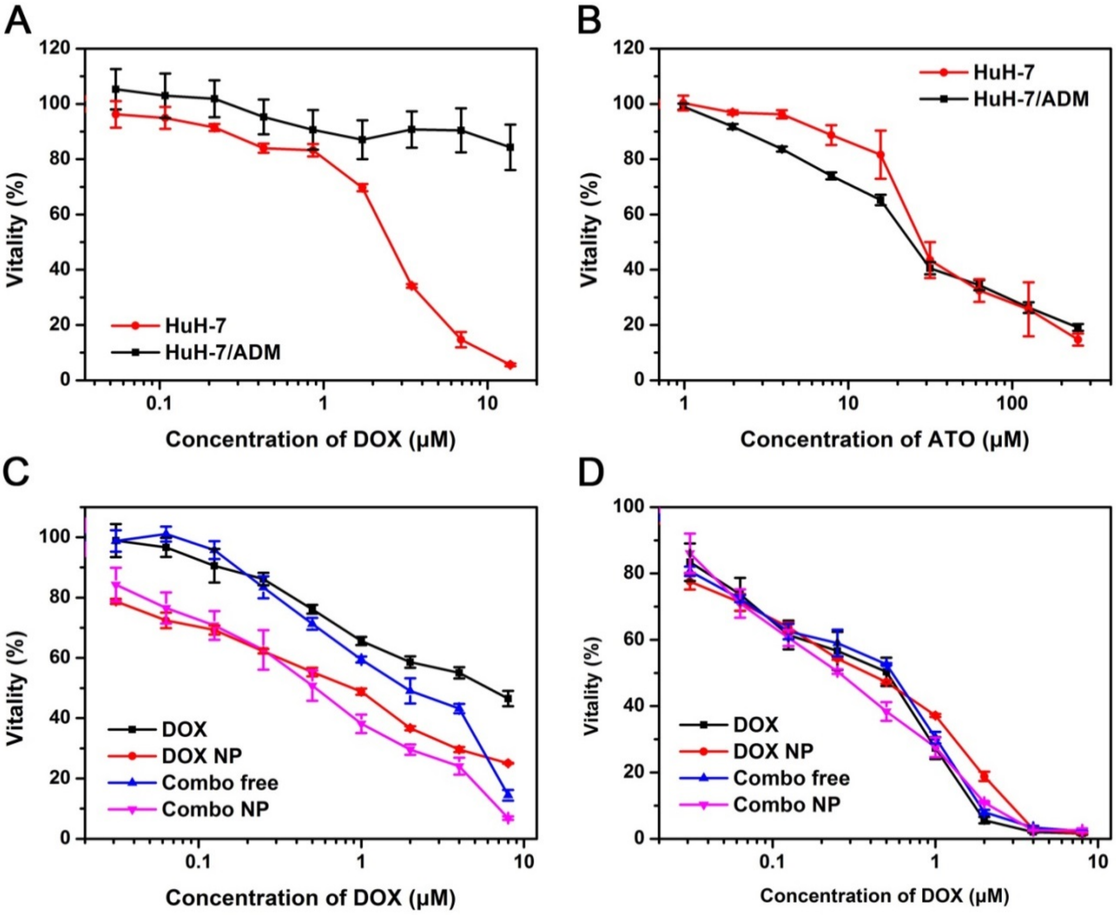

Supplementary Figure 2. The vitality analysis of HuH-7 and HuH-7/ADM cells treated with (A) DOX and (B) ATO. Vitality of (C) HuH-7/ADM and (D) HuH-7 after treated with different drug formulations at various DOX concentrations for 48 h. All data are represented as average ± standard deviation (n = 5).

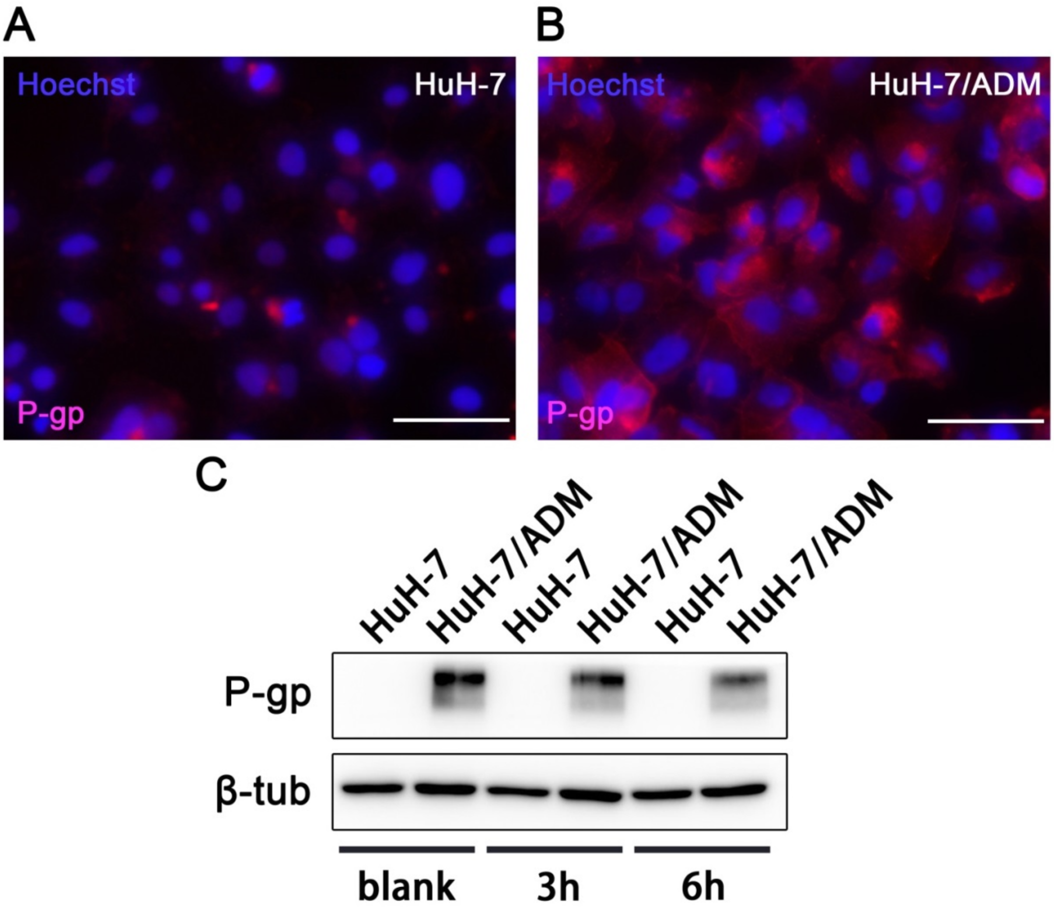

Supplementary Figure 3. The expression of P-gp in (A) HuH-7 and (B) HuH-7/ADM cells by immunofluorescence and (C) western blotting. Cells were treated with 1 μM DOX for 3 or 6 h. Scale bars: 50 μm. The P-gp was overexpressed around the cell membrane of HuH-7/ADM cells.

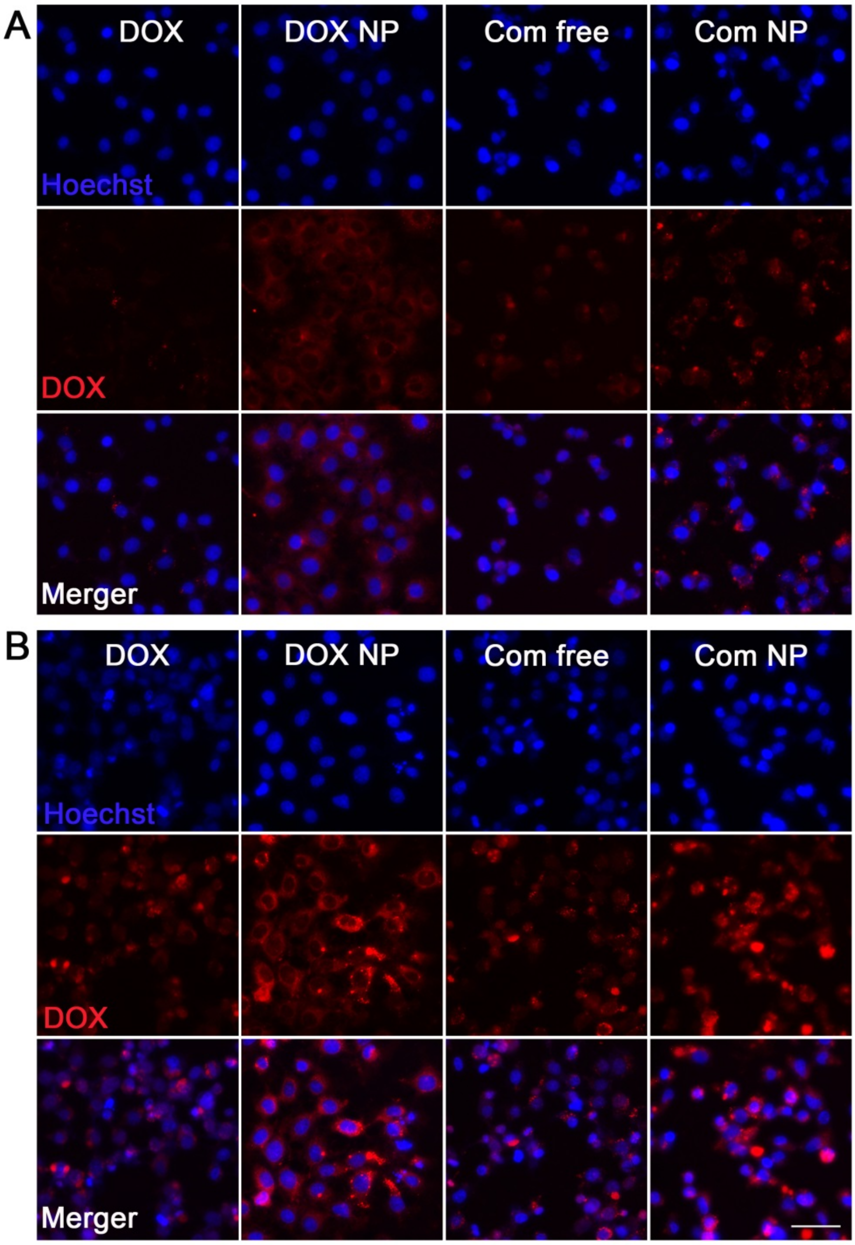

Supplementary Figure 4. Intracellular localization of DOX after treated with different drug formulations for 12 h and observed by fluorescence microscope. (A) HuH-7/ADM and (B) HuH-7 cells treated with 4 μM DOX, DOX NP, Combo free and Combo NP for 12 h, scale bar: 50 μm. Hoechst 33342 was used to stain cell nuclei.

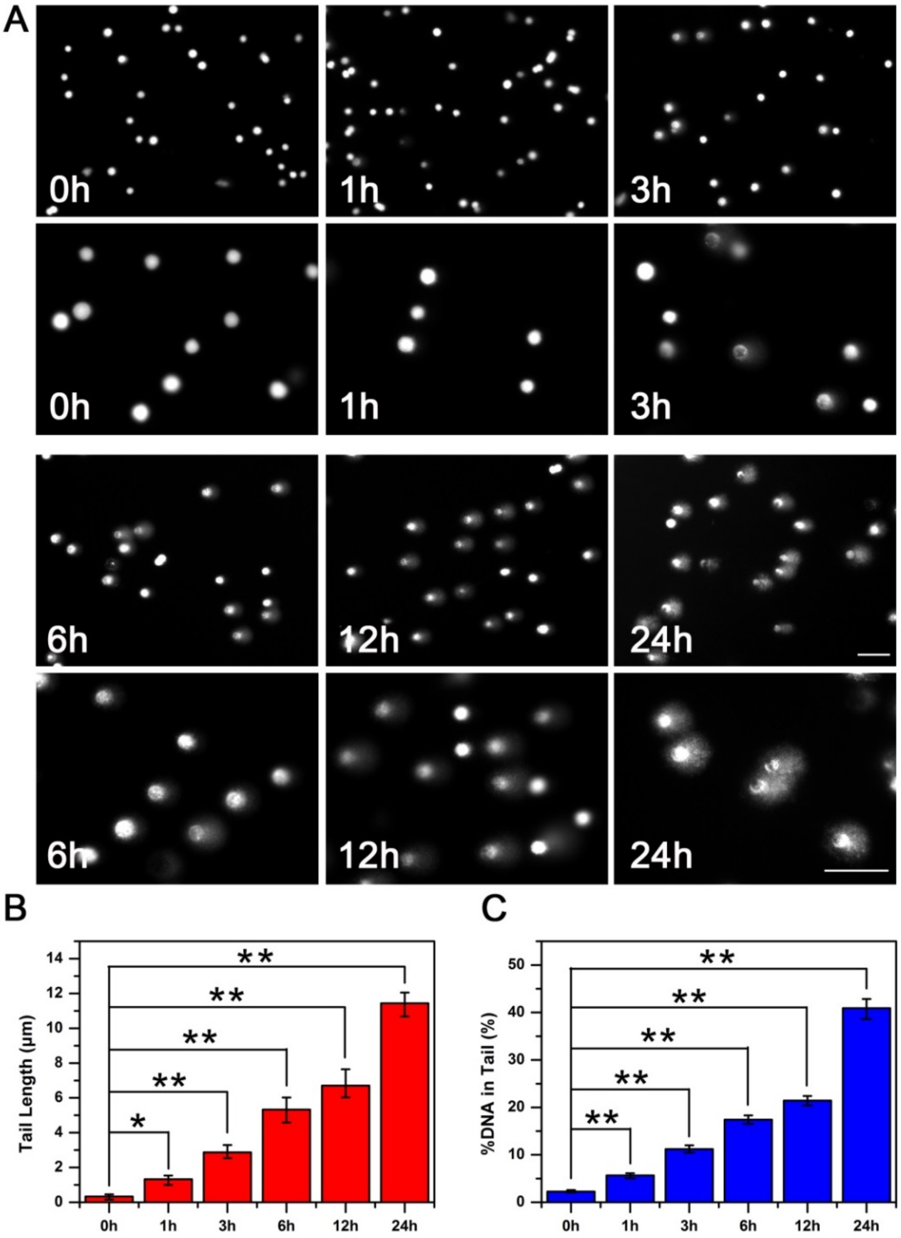

Supplementary Figure 5. Comet assay analysis for DNA damage. (A) Fluorescence microscope imaging of HuH-7 cells treated with 2 μM DOX in different times, and the two pictures (upper and lower) were obtained by the same experiment with different magnifications. DNA was stained by PI, scale bar: 50 μm, (B) The tail length and (C) the percentage of DNA in tail were analyzed for 200 cells at random by CometScore software. All data are represented as average ± standard deviation (n = 3), *p < 0.05; **p <0.01. DOX could cause DNA damage of HuH-7 cells in a time-dependent manner.

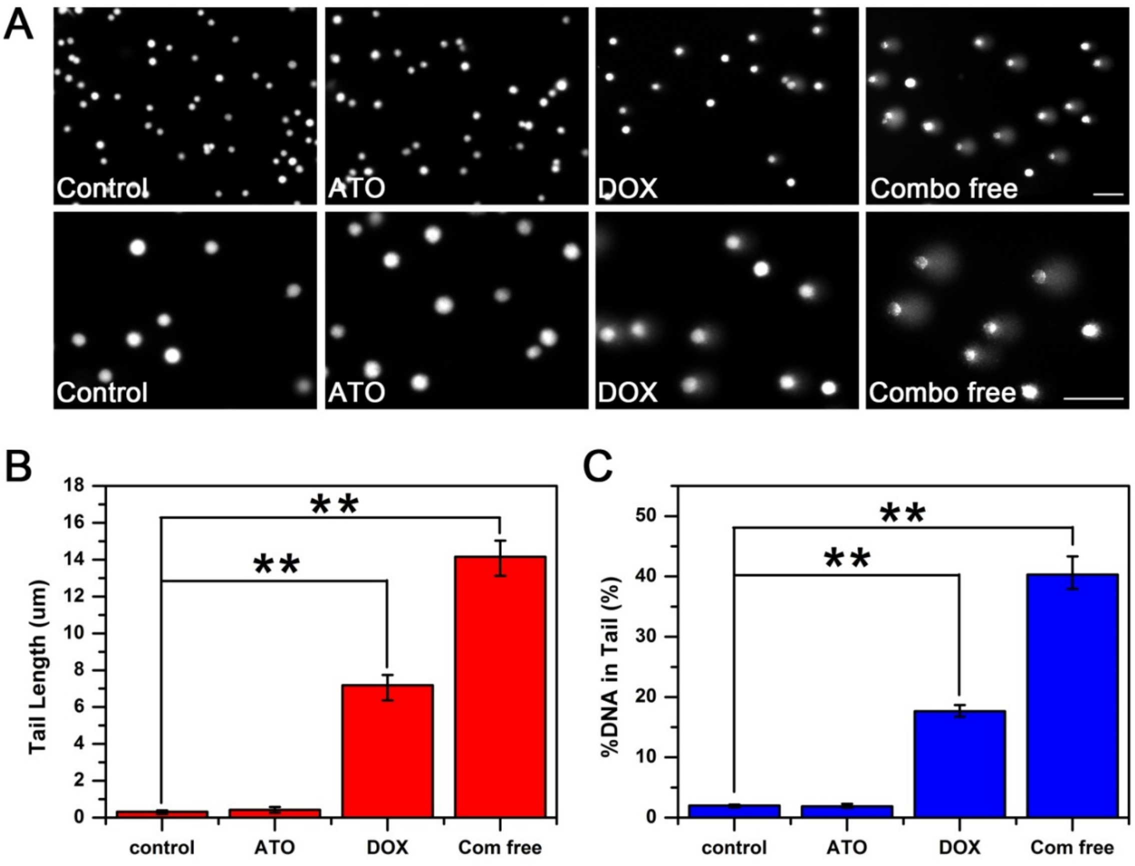

Supplementary Figure 6. Comet assay analysis for DNA damage. (A) Fluorescence microscope imaging of HuH-7 cells treated with different drug formulations (2 μM DOX and 4 μM ATO) for 12 h, and the two pictures (upper and lower) were obtained by the same experiment with different magnifications. DNA was stained by PI, scale bar: 50 μm. (B) The tail length and (C) the percentage of DNA in tail were analyzed for 200 cells at random by CometScore software. All experiments are represented as average ± standard deviation (n = 3), $**p < 0.01$. ATO can significantly enhance the DNA damage induced by DOX in HuH-7 cells.

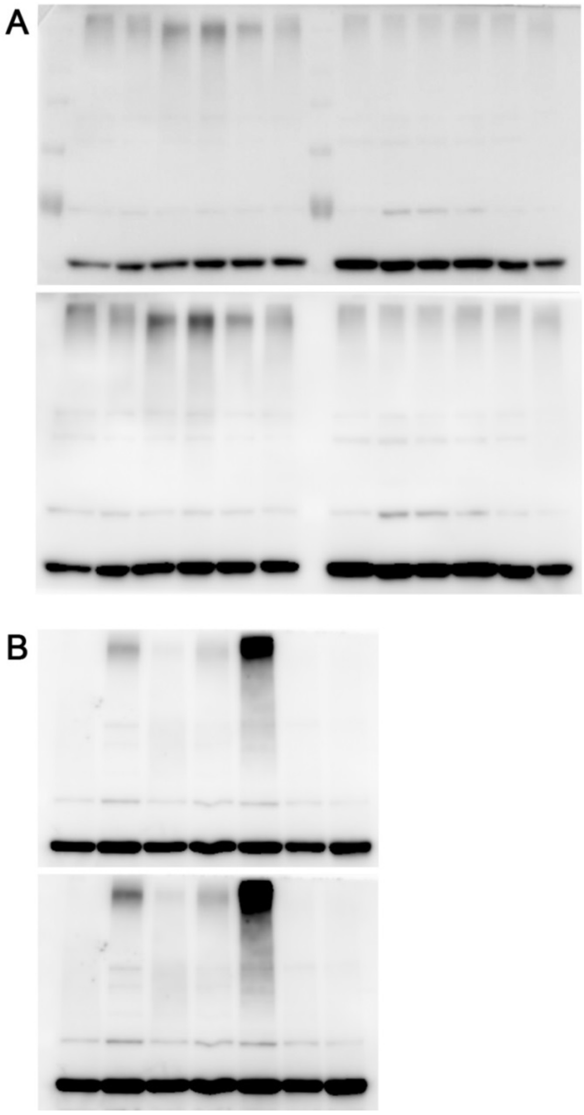

Supplementary Figure 7. Full-length blots of Fig. 5 with multiple exposures. (A) HuH-7 and HuH-7/ADM cells treated with 4 μM ATO for different incubation times (upper: short exposure time, lower: long exposure time). (B) HuH-7/ADM cells were treated with different drug formulations (2 μM DOX and 4 μM ATO) for 12 h (upper: short exposure time, lower: long exposure time).

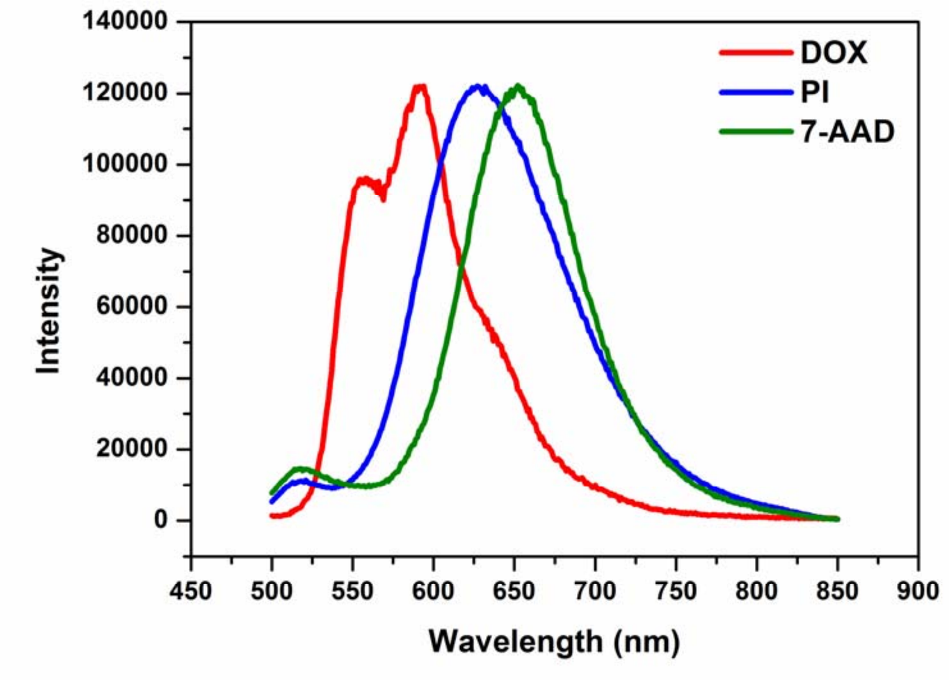

Supplementary Figure 8. The emission spectra of DOX, PI, and 7-AAD after 488 nm excitation. The overlap of DOX and 7-AAD is less than that of DOX and PI, indicating that 7-AAD is a better candidate as staining dyes of necrotic cells for flow cytometry and apoptosis analysis in this work.
